# Cactus-like Metamaterial Structures for Electromagnetically Induced Transparency at THz frequencies

**DOI:** 10.1021/acsphotonics.4c01179

**Published:** 2024-10-07

**Authors:** Savvas Papamakarios, Odysseas Tsilipakos, Ioannis Katsantonis, Anastasios D. Koulouklidis, Maria Manousidaki, Gordon Zyla, Christina Daskalaki, Stelios Tzortzakis, Maria Kafesaki, Maria Farsari

**Affiliations:** † Institute of Electronic Structure and Laser, Foundation for Research and Technology—Hellas (FORTH-IESL), GR-70013 Heraklion, Crete, Greece; ‡ Department of Physics, National and Kapodistrian University of Athens, GR-15784 Athens, Greece; § Theoretical and Physical Chemistry Institute, National Hellenic Research Foundation, GR-11635 Athens, Greece; ∥ Department of Physics and Regensburg Center for Ultrafast Nanoscopy (RUN), University of Regensburg, 93040 Regensburg, Germany; ⊥ Department of Materials Science and Engineering, University of Crete, GR-70013 Heraklion, Crete, Greece

**Keywords:** 3D metamaterials, electromagnetically induced transparency, direct laser writing, THz sources, broken symmetry, quasi-dark resonances

## Abstract

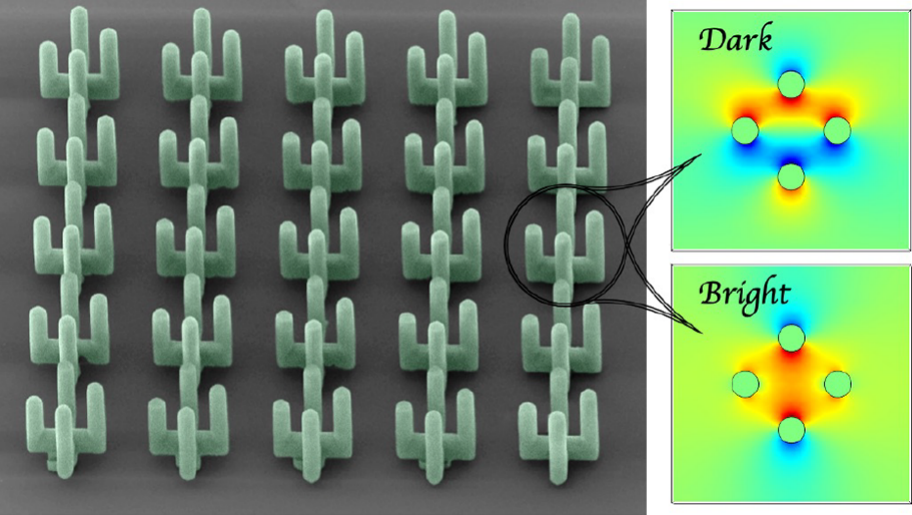

THz metamaterials present unique opportunities for next-generation technologies and applications as they can fill the “THz gap” originating from the weak response of natural materials in this regime, providing a variety of novel or advanced electromagnetic wave control components and systems. Here, we propose a novel metamaterial design made of three-dimensional, metallic, “cactus-like” meta-atoms, showing electromagnetically induced transparency (EIT) and enhanced refractive index sensing performance at low THz frequencies. Following a detailed theoretical analysis, the structure is realized experimentally using multiphoton polymerization and electroless silver plating. The experimental characterization results obtained through THz time domain spectroscopy validate the corresponding numerical data, verifying the high potential of the proposed structure for slow light and sensing applications.

## Introduction

Terahertz (THz) radiation technology, which aims to bridge the realms of electronics and photonics, has garnered significant interest over the past 2 decades. THz waves possess distinct features, such as their nonionizing nature, ability to penetrate many nonconductive materials, and capability to directly probe molecular vibrational and rotational modes. These unique characteristics have led to extensive use in various applications, including medical diagnostics, industrial quality control, security scanning, and fundamental physics research.^1−3^ Moreover, THz waves are pivotal in advancing THz wireless communication systems, particularly within the rapidly evolving landscapes of 5G and 6G technologies.^4^ In this context, they offer substantial benefits by providing increased bandwidths to meet the high data demands of next-generation communication networks.

In the framework of these applications, the phenomenon of slow light is a significant advantage. Slow-light media can exhibit very low propagation velocity of electromagnetic waves^5^ or even stop light entirely.^6^ This enhances light-matter interactions, leading to improved sensitivity and resolution in imaging, sensing, and communication and facilitating the significant miniaturization of photonic devices.^7−9^ To achieve this slow-light effect, one effective method is electromagnetically induced transparency (EIT).^10−12^ EIT is a quantum interference phenomenon that makes an otherwise opaque medium transparent in a narrow frequency window with low absorption and sharp dispersion.^13^ This effect not only slows down the group velocity of light but also facilitates significant optical effects by controlling the medium’s electronic quantum states.

In recent advancements, the realization of EIT at THz frequencies has been successfully achieved by using metamaterial structures. Metamaterials, composed of subwavelength resonant components (meta-atoms),^14^ allow for the manipulation of electromagnetic waves across a broad frequency spectrum, often in unconventional ways,^15^ alternating the electromagnetic properties of conventional materials by giving a specific geometry. These materials initially demonstrated exotic optical properties in structures composed of simple metallic and/or dielectric materials,^16−19^ and recent advancements incorporate complex media, yielding even more extraordinary responses, such as those observed in quantum systems.^20−22^ These engineered materials can be designed to support resonances that mimic the EIT phenomenon, enabling slow-light effects. The characteristic features of EIT, simultaneously low absorption and sharp dispersion, can be observed in classical systems with coupled resonators^23,24^ and described by a simple model of two coupled harmonic oscillators resulting in dark and bright resonances^25^ which are excited by breaking the symmetry of the metamaterial, creating states which otherwise would be forbidden.

Most experimental demonstrations of EIT-like response with metamaterials have been performed with planar meta-atom structures.^12,26,27^ Using three-dimensional meta-atoms instead can prove advantageous for several applications.^28−30^ For instance, their larger surface area can lead to an increased light-matter interaction. In sensing applications, the analyte can occupy a large volume surrounding the 3D meta-atom and interacting with the strong local fields, thus leading to enhanced sensitivity. Note that a sensing application is also naturally suited to the sharp spectral feature of the EIT-like response; not only the sensitivity but also the sensitivity over the line width ratio (a typical figure of merit in sensing systems) would be enhanced. In addition, 3D meta-atoms allow for more design flexibility in breaking the symmetry with respect to mirror planes of the structure. This can be useful for introducing controllable coupling to already existing dark resonant modes. However, fabrication of 3D meta-atoms is challenging with standard lithographic techniques, such as photolithography, e-beam lithography, or nanoimprint lithography.^31^ Fortunately, multiphoton polymerization (MPP) offers the unique advantage to realize elaborate 3D structures with practically arbitrary complexity. Focusing in low THz frequencies, the subwavelength components have to be a few μm in size. Adding the 3D geometry to the complexity of the structure, MPP enables the fabrication of these kinds of structures, while the optical, chemical, and mechanical properties of the photosensitive materials can be modified according to the requirements of the study. Using MPP also provides the capability for further postprocessing techniques such as calcination and metallization process.

In this paper, we exploit a 3D metallic “cactus-like” structure to trigger the EIT phenomenon in low THz frequencies (0.1–10 THz) and demonstrate its potential for advanced applications in slowing light and environmental refractive index sensing, going one step forward in filling the “THz gap” with new applications revolving around light-matter interaction. The proposed structure is composed of a two-dimensional array of paired, free-standing vertical U-shaped ring resonators,^32^ featuring an asymmetry in one arm of each U-pair. The asymmetry takes place in the *yz* plane, and when the sample is excited with linearly polarized light along the *y*-axis (propagating in the *z*-direction), the quasi-dark appears; its interference with the bright resonance results in a sharp transmission peak within a broad transmission dip. In this study, an extensive explanation of how the EIT phenomenon is triggered in this specific design is elaborated by using numerical simulations and a multipole decomposition of the induced conduction current in the structure. Moreover, a simple resistor–inductor–capacitor (RLC) model is provided, able to explain and reproduce not only the response of our structure but also those of many different systems where broken symmetry induces sharp EIT-like features.

The uncommon electromagnetic response of our structure results in a steep change in the transmission phase over a narrow frequency window, leading to high values of group delay and allowing delay of an incident beam by up to ∼2200 optical cycles. In addition, due to the metallic properties and the topology and sensitivity of the resonant modes, our structure shows advanced sensitivity in variations of the refractive index of the environment, being able to reach figures of merit as high as 34. The theoretical design is translated into a real-life structure using MPP as a fabrication tool and subsequently electroless silver plating to obtain the metallic metamaterial. The results of the theoretical analysis for a specific value of cut in the asymmetry of the “cactus-like” design are validated using THz-time domain spectroscopy (THz-TDS), demonstrating the triggering of EIT response in the proposed metamaterial.

## Results

### “Cactus” Meta-Atom and Supported Resonances

**Figure 1:**
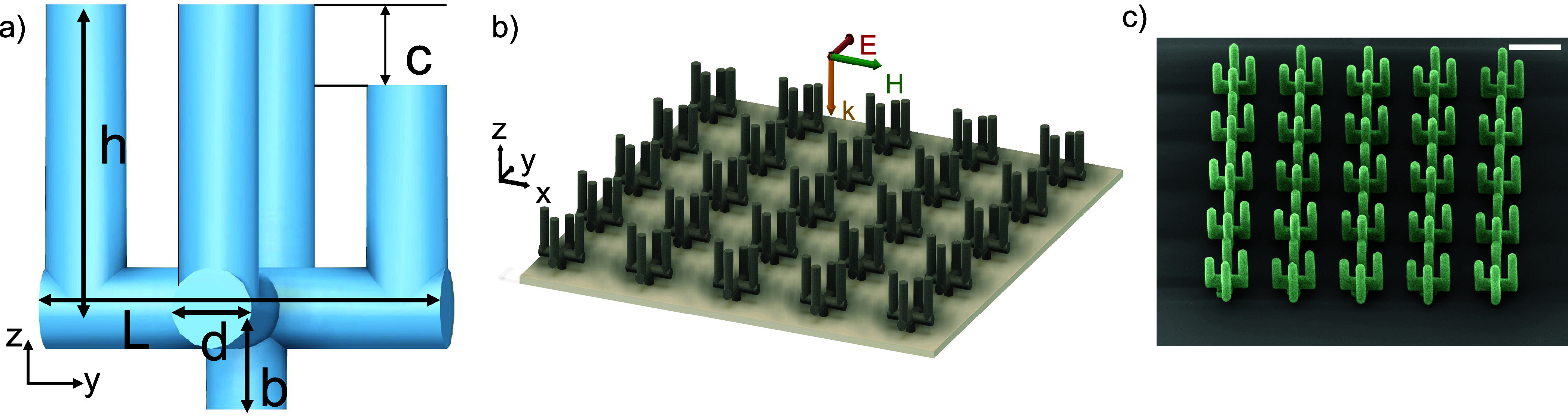
Schematic of the proposed metamaterial structure (metasurface). (a) Unit cell with a “cactus-like” meta-atom. One arm can be shorter by a value of *c*. (b) Metasurface composed of multiple meta-atoms. The incident field is a *y*-polarized normally incident plane wave. (c) Artificial SEM image of the cactus structures presented for visual purposes. Green color represents the cactus metamaterial, and the scattering effect from silver nanoparticles is visualized (scale bar 50 μm).

The proposed 3D “cactus-like” geometry is illustrated in Figure 1a. It consists of a periodic arrangement (square lattice in the *xy* plane) of 3D meta-atoms [Figure 1b,c]. Each meta-atom is formed by two metallic, vertically positioned U-shaped split-ring resonators (SRRs) placed in the *xz* and *yz* planes. In addition, one of the two SRRs (the one in the *yz* plane) can be asymmetric, when one vertical arm is cut and shorter by a value *c* from the other one. This specific microstructure offers the ability to control all important parameters of symmetric and asymmetric features, and it covers a large volume area of the unit cell, which is crucial for sensing applications since the light-matter interaction is increased. The asymmetric features give full control of the EIT phenomenon and, subsequently, the group delay and sensitivity of the metamaterial. The metallic behavior of the structure offers enhanced interaction with THz radiation and higher sensitivity to refractive index changes. These two characteristics of geometry and material properties are critical parameters to excite EIT in low THz frequencies. The metal that was used is silver which provides high conductivity, and it is suitable for low THz frequencies regarding the light-matter interaction.

**Figure 2:**
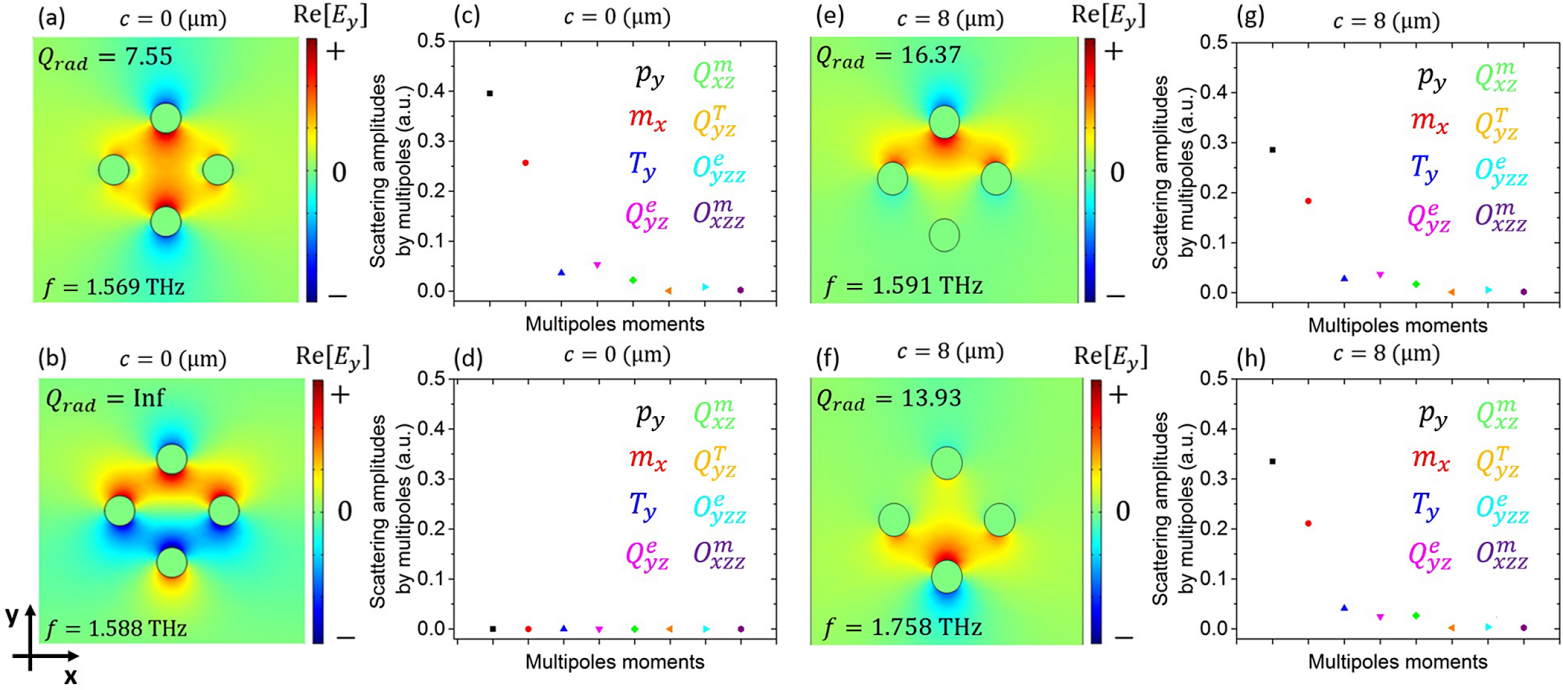
Bright and dark eigenmodes supported by the “cactus” meta-atom. (a,b) Electric field distribution (*E*_*y*_ component) at a *xy* plane bisecting the meta-atom for the (a) bright and (b) dark modes in the symmetric *c* = 0 case. The resonant frequencies and *Q*_rad_ values are also included. (c,d) Multipole decomposition of the conduction current distribution in the meta-atom, indicating the contribution of each multipole moment to the *y-*polarized scattered field. In the dark mode [panel (d)], all contributions are near-zero. (e,f) Same as panels (a,b) when the meta-atom becomes asymmetric with *c* = 8 μm. (g,h) Same as panels (c,d) when *c* = 8 μm. The second mode becomes quasi-dark and now exhibits considerable *p*_*y*_ and *m*_*x*_ components.

Starting our investigation from the symmetric meta-atom (no cut, *c* = 0) and solving an eigenvalue problem of the periodic unit cell to determine the resonant modes of the metasurface. For the simulations, it was considered a 500 nm silver coating layer with conductivity σ_Ag_ = 6.3 × 10^6^ S/m. The dimensions of the meta-atom are *h* = 40 μm, *L* = 40 μm, and *d* = 9 μm, and the unit cell size is 80 μm. The substrate and support leg are momentarily omitted to simplify the structure and focus on the underlying physics, concentrating on the two modes, which are depicted in Figure 2a,b. Besides the electric field distribution (*E*_*y*_ component), the resonant frequencies and radiation quality factors, *Q*_rad_, are also included. It can be seen that the modes lie close in frequency, but they exhibit quite different *Q*_rad_ values. The mode in Figure 2a is bright, i.e., it possesses a finite and relatively low *Q*_rad_ and can be directly accessed with a *y*-polarized normally incident plane wave. This is corroborated by the even parity of the *E*_*y*_ component with respect to the *xz* and *yz* planes. On the other hand, the mode in Figure 2b is dark; it possesses a practically infinite *Q*_rad_ and cannot couple to a normally incident *y*-polarized plane wave. This is corroborated by the odd parity exhibited by the *E*_*y*_ component with respect to the *xz* plane.

Next, we investigate further the electromagnetic character of each mode by performing a multipole decomposition.^33^ The far-field contribution of each multipole to a *y-*polarized scattered field^34^ is presented in Figure 2c,d for the two modes under study. In the bright-mode case (Figure 2c), we see strong electric (*p*_*y*_) and magnetic (*m*_*x*_) dipole contributions, indicating that both the *E*_*y*_ and *H*_*x*_ components of a *y*-polarized plane wave can excite the eigenmode. In addition, we find non-negligible contributions by the electric quadrupole *Q*_*yz*_^*e*^ and toroidal dipole *T*_*y*_ since the finite dimensions and elaborate geometry of the meta-atom lead to a more complex near-field structure of the resonance. In sharp contrast, for the dark mode (Figure 2d), we find near-zero contributions to a *y*-polarized scattered field for all multipole moments. This is yet another confirmation of the dark nature of the mode.

We now examine the role of asymmetry by introducing a cut of *c* = 8 μm in one of the arms (see Figure 1a). The frequencies of the two modes shift, as anticipated, and the new resonant frequencies are *f* = 1.591 and *f* = 1.758 THz. More importantly, due to the broken symmetry, the dark mode now becomes quasi-dark.^35^ This strategy has received renowned interest recently in the context of quasi-bound states in the continuum.^36^ Specifically, the mode profile is not strictly antisymmetric with respect to the *xz* plane anymore, the radiative quality factor becomes finite (*Q*_rad_ ∼ 13.93 in this case), and excitation via a normally incident *y-*polarized plane wave is allowed. Note that the symmetry with respect to the *yz* plane has not been disturbed. As discussed in the following, the excitation of the quasi-dark mode introduces a sharp transmission peak within a broad transmission dip, leading to a spectral response reminiscent of electromagnetically induced transparency. This significant change in the radiative characteristics is imprinted in the multipole expansion as well (Figure 2f); the *p*_*y*_ and *m*_*x*_ contributions are not negligible anymore and can mediate coupling with an incoming *y*-polarized plane wave (e.g., incident wave or local *E*_*y*_ and *H*_*x*_ fields produced by the originally bright mode). Slight changes in the multipole composition are also seen for the bright mode (Figure 2e). In the Supporting Information (SI), we track the evolution of the multipole contributions with varying *c*, providing also the corresponding frequencies and Q-factors.

### EIT Response and Sensing Performance

#### EIT Response

**Figure 3:**
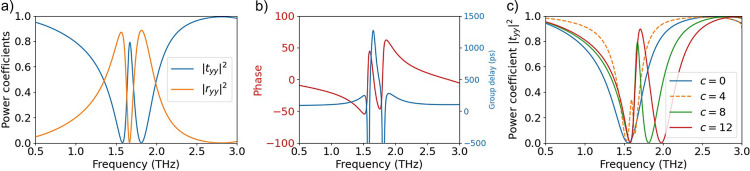
Simulated results of the electromagnetic response for the proposed structure for *y*-polarized light. (a) Reflection (red curve) and transmission (blue curve) power coefficients (*R*_*yy*_, *T*_*yy*_) for asymmetry parameter *c* = 8 μm. (b) Transmission phase (red curve) and group delay (blue curve) for *c* = 8 μm. (c) Transmission (power) coefficient for different values of *c*; the EIT feature arises when *c*≠ 0.

We next calculate plane-wave scattering coefficients for the metasurface under study by means of full-wave simulations based on the finite element method. We assume that a *y*-polarized plane wave impinges on the metasurface at normal incidence. Transmission and reflection coefficients for the *c* = 8 μm case are depicted in Figure 3a.

As discussed in Figure 2f, in the asymmetric structure (*c*≠ 0), the quasi-dark mode can be excited. It interferes with the bright mode, and as a result, a sharp transmission peak appears within the broad transmission dip; the quasi-dark mode resonant frequency emerges at *f* ∼ 1.758 THz. The interference between the two modes is further discussed in the SM by performing a multipole expansion on the induced conduction current and identifying that both the bright and quasi-dark modes are characterized predominantly by *p*_*y*_ and *m*_*x*_ contributions (cf. Figure 2). Note that in the symmetric structure (*c* = 0), the antisymmetric mode is completely dark, and a single conventional transmission dip appears associated with the bright resonance (see SI). In the same way, when the incident wave is *x*-polarized, the quasi-dark mode cannot be excited and a single transmission dip appears in the scattering coefficients (see SI).

The response in Figure 3a is reminiscent of the quantum phenomenon of EIT and is, thus, frequently termed “photonic analogue of EIT.”^24,37^ This analogy is further corroborated by looking at the transmission phase in Figure 3b. Indeed, there is a central region of steep phase delay with a negative slope. This region can be used for delaying light,^38^ as can be seen by the group delay which is calculated as τ_g_ = −*d*ϕ(ω)/*d*ω and reaches a value of 1.3 ns (approximately 2200 times the carrier cycle at 1.7 THz). The characteristics of the EIT feature can be readily controlled by varying the degree of asymmetry (parameter *c*). This is depicted in Figure 3c. As *c* increases, the quasi-dark mode becomes brighter, and the EIT peak becomes broader. At the same time, the maximum group delay decreases, but the bandwidth that can be used for pulse delaying purposes increases.

#### Equivalent Model of Coupled RLC Circuits

**Figure 4:**
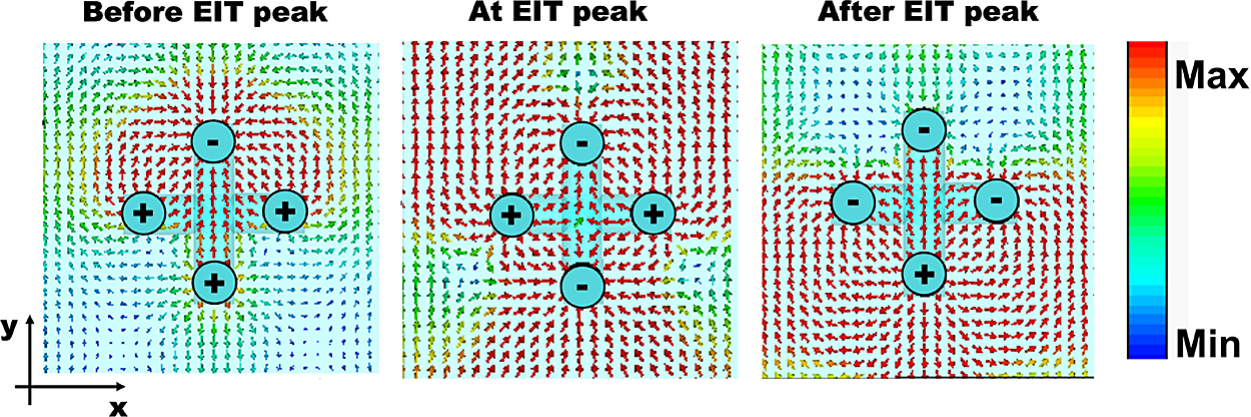
Electric field at the transmission dip just before the EIT peak of our structure (left-panel), at the EIT peak (center), and at the dip just after EIT peak (right-panel) at a *x*–*y* plane close to the top of the structure. The accumulated charge, as determined from the fields, is also marked. The shortened vertical arm of the structure is the top one.

To gain additional insight into the EIT response of our structure, we derive an “equivalent” model based on coupled RLC resonant electrical circuits. This allows to obtain approximate expressions for the structure polarizabilities and, through them, for the transmission and reflection amplitudes.^39^ To derive an appropriate RLC model for our structure, it is helpful to examine the charge and current distributions at the EIT peak and at the transmission dips immediately before and after it. For *c* = 8 μm, these distributions are depicted in Figure 4, which shows the electric field at the three frequencies.

**Figure 5:**
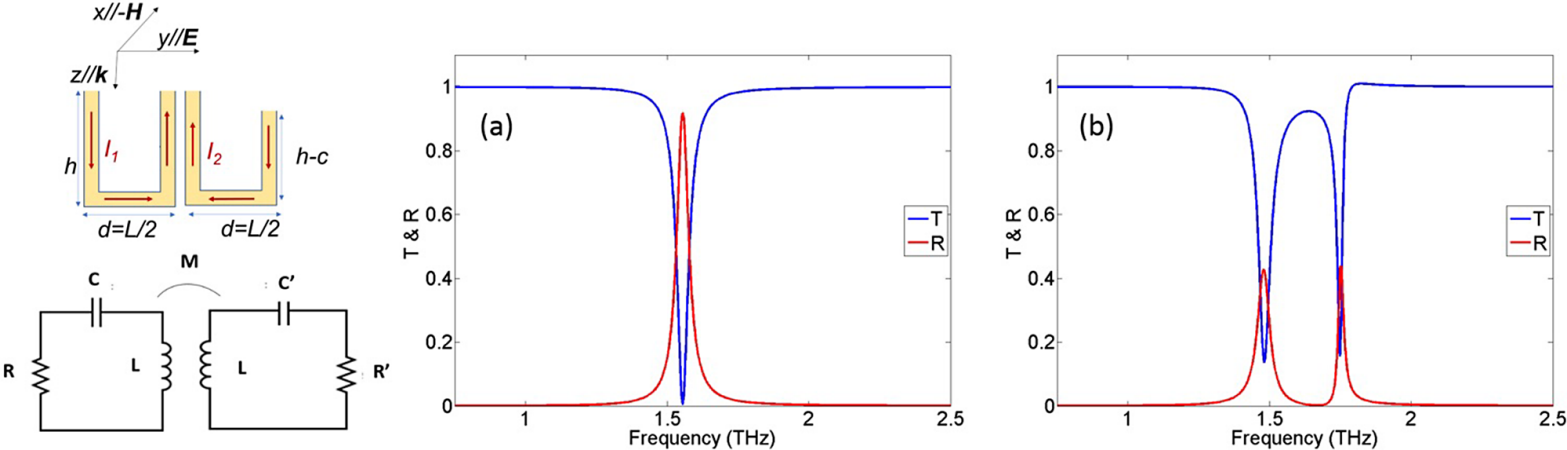
Left panel: A simple two-resonator system approximates the electromagnetic response of the cactus structure. The geometrical parameters are as in the actual structure (see Figure 1). The direction of currents *I*_1_ and *I*_2_ corresponds to the dark mode. Transmitted and reflected power were obtained using the analytic expressions stemming from the RLC model. Panels (a,b) correspond to the symmetric (both U-rings the same) and asymmetric (one U-ring-arm shorter) cases of our system, respectively. The following parameters were used: *d* = 40 μm, *A* = 1.6 × 10^–10^ m^2^, *L* = 1.5 × 10^–10^ H, *C* = 0.065 fF, *R*_0_ = 50 Ω, δ*R* = 1.25 Ω, M = 0.075*L*, and *A*_uc_ = 6.4 × 10^–9^ m^2^.

As can be seen in Figure 4, the transmission dips align with the resonant responses of the top and bottom halves of the structure. At the EIT peak, looplike (antiparallel) currents are observed in the neighboring cactus arms, forming four current loops. Due to the structure’s mirror symmetry with respect to the *xz* plane bisecting the structure, these currents result in the vanishing of the total induced moments *p*_*x*_ and *m*_*y*_. However, the asymmetry with respect to the *yz* plane leads to nonvanishing *p*_*y*_ and *m*_*x*_, as confirmed also by the multipole analysis. A detailed examination of the loop currents and their induced electric and magnetic dipole moments reveals that the structure can be effectively described as its projection in the *yz* plane (including both the geometry and the fields/currents), resembling two slightly dissimilar coupled U-shaped resonators. Figure 5 provides a simplified illustration of these resonators: two coupled U-rings with one having a slightly shorter vertical arm. As we will demonstrate below, shortening one of the four vertical arms of the structure (thus breaking its mirror symmetry) excites a quasi-dark mode, characterized by opposite loop currents in the two rings (see arrows *I*_1_ and *I*_2_ in Figure 5). The coupling of this quasi-dark mode with the original bright mode, characterized by parallel loop currents in the U-rings, is directly proportional to the dissimilarity of the two rings.

Considering excitation of the structure of Figure 5a by a *y*-polarized plane wave, the circuit equations describing the currents in the two rings can be written as
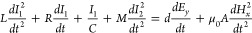
1
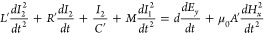
2where subscripts 1 and 2 refer to the symmetric and asymmetric rings, respectively, *I*_1,2_ are the currents, *L* and *L′* are the inductances, *R* and *R′* are the resistances, *C* and *C′* are the capacitances, *A* and *A′* are the loop areas of each ring, and *M* is the coupling coefficient (primed quantities refer to the asymmetric ring). In the following, for simplicity of the analytical formulas, we consider *A* = *A′* and *L* = *L′*. Moreover, we express in the above equations the resistances and capacitances as *R* = *R*_0_ + δ*R*, *R′* = *R*_0_ – δ*R*, 1/*C* = 1/*C*_0_ – δ(1/*C*), and 1/*C′* = 1/*C*_0_ + δ(1/*C*), where *R*_0_ and 1/*C*_0_ are the average resistance ((*R* + *R′*)/2) and average inverse-capacitance, respectively. Considering the harmonic time dependence of the form *e*^–*i*ω*t*^, it is straightforward to show that on adding and subtracting the result from eqs 1 and 2, the differential equations one can arrive at are
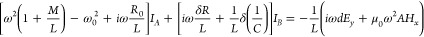
3
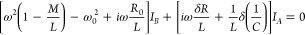
4with *I*_*A*_ = (*I*_1_ + *I*_2_)/2, *I*_*B*_ = (*I*_1_ – *I*_2_)/2, and ω_0_ = 1/*LC*_0_.

Equations 3 and 4 show that breaking the mirror symmetry in the double U-ring structure of Figure 5a results in a coupled system of a bright and a dark resonator, i.e., a system capable of exhibiting an EIT response. The dark resonator current is maximized for *I*_2_ = −*I*_1_, i.e., for equal antiparallel currents in the two U-rings (see the current configuration in Figure 5a). The coupling of the two resonators (bright and dark), strongly related to the quality factor of the EIT peak, is directly proportional to the dissimilarity of the two U-rings coming from the broken symmetry (i.e., the U-arm shortening). If the cut *c* of the broken-symmetry-ring is zero, then *I*_*B*_ = 0 ⇒ *I*_1_ = *I*_2_, and the only resonance that is excited is the bright one, corresponding to a single U-ring magnetic resonance, slightly shifted due to the U-rings’ mutual coupling. Having shown the equivalence of eqs 1 and 2 with a dark-bright coupled resonator system, we can proceed using eqs 3 and 4 and calculate the induced electric and magnetic dipole moments (produced by the current *I*_*A*_, the only one that radiates). Through them, we can specify the polarizabilities and the transmission and reflection coefficients, as discussed in the Supporting Information.

The RLC model presented above is mainly qualitative, aiming to provide insight into the electromagnetic response of our system and assess its dependence on the system parameters, in particular on the asymmetry. However, it can also approximate quite well the numerical results for the transmitted and reflected power, *T* = |*t*|^2^ and *R* = |*r*|^2^. In Figure 5, we plot transmitted and reflected power calculated with the analytic expressions for the symmetric (i.e., identical U-rings) and asymmetric cases of our two U-ring system. As can be observed, the analytic model and numerical simulation (see Figure 3c) are in very good agreement.

#### Sensing Performance

**Figure 6:**
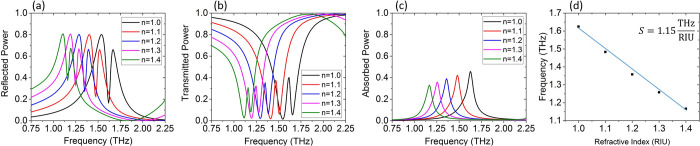
Sensing performance of the proposed metamaterial for different values of RI. (a) Reflected power, (b) transmitted power, (c) absorbed power, and (d) tracing of the resonance shift for RI variation from *n* = 1 to *n* = 1.4.

Next, we investigate the merits of the proposed structure as a refractive index (RI) sensor. As mentioned in the introduction, the 3D meta-atom allows for increased surface area for light-matter interaction.^40^ We choose a small degree of asymmetry (*c* = 4 μm) so that the EIT peak is sharp. We assume that the analyte exactly covers the meta-atoms and conduct simulations for a varying RI value for the analyte in the range *n* = 1–1.4 (relevant for biological media). The reflection, transmission, and absorption coefficients are depicted in Figure 6a–c, respectively. The sharp EIT feature shifts linearly as the RI of the analyte changes, and its position in either transmission or reflection can be readily traced. This linear shift is shown in Figure 6d. From the slope, we can deduce a sensitivity value of *S* = Δ*f*/Δ*n* = 1.15 THz/RIU.^41^ This high sensitivity can also be attributed to the strong local fields and the 3D nature of the metallic “cactus” meta-atom.^40^ In addition, to better assess the sensing performance, we introduce the typical figure of merit (FOM = *S*/fwhm), which takes into account the line width of the EIT feature. The full width at half-maximum (fwhm) is determined via the transmission curve. For the *c* = 4 μm case studied in Figure 6, the FOM is calculated to be 34 which is higher than what recent works have shown in electromagnetic metamaterials operating in THz frequencies in combination with the enhanced sensitivity.^12,42−44^

The results in Figure 6 demonstrate a wide detection range of Δ*n* = 0.4 with a constant slope (sensitivity) that can be used for different aqueous and gas molecules. In the upper range of RIs, the proposed highly sensitive sensor can be used to detect the biological material, such as proteins (*n* = 1.4), muscle (*n* = 1.39), skin (*n* = 1.36), and blood plasma (*n* = 1.335), as well as cell culture medium PBS (*n* = 1.31–1.34), with high accuracy. This can be extended to liquids, such as kerosene (*n* = 1.39), acetone (*n* = 1.36), and water (*n* = 1.33). In lower RIs, the sensor can be used for the detection of chemical liquefied greenhouse gases such as liquid helium (*n* = 1.04), liquid hydrogen (*n* = 1.11), liquid oxygen (*n* = 1.22), and liquid methane (*n* = 1.29). We have also investigated the performance of RI sensing for higher indices in the range *n* = 1.5–1.8 (see SI). In this case, we obtain a sensitivity value of *S* = 0.825 THz/RIU, which shows that our sensor can also be used for analytes with higher RIs.

The comparison between the proposed metamaterial and different sensors in the literature is included in Table 1. In most cases, the performance of our design is superior. Additionally, the proposed 3D exhibits greater design flexibility via the multiple geometric degrees of freedom and the ability to tailor the symmetries along all three Cartesian axes. This enables fine control over the system response and the opportunity for supporting broken-symmetry quasi-dark resonances.

**Table 1:** Comparison of Relevant Metrics of *Q*-Factor, Sensitivity (*S*), and FOM of Different Sensor Designs at THz Frequencies

refs	*Q*	*S* (THz/RIU)	FOM
(45)	84.07	1.12	50.69
(46)		0.07	3
(42)	175	0.598	
(43)	63	0.096	7.6
(47)		59.24	18.1
(48)	22.50	0.3	2.94
(49)	14.3	0.28	4
(50)	120	0.187	19.1
this work	31.34	1.15	34

### Fabrication of “Cactus” Metamaterial and Experimental Verification of EIT

The experimental validation of the proposed metamaterial is realized using MPP. Given its nonlinear characteristics, MPP enables true and maskless 3D printing with submicron resolution, a capability highly beneficial for various tasks across interdisciplinary research areas.^51−58^ These characteristics make MPP an ideal tool for the fabrication of metallic “cactus-like” resonators with both symmetric and asymmetric designs and for experimentally verifying their optical properties. Although MPP mainly facilitates the processing of dielectric structures, the use of a photoresist containing a precursor with moieties capable of binding metals enables the selective metallization of MPP-processed structures in a postprocessing step. This metallization process is achieved via a highly selective chemical approach known as silver electroless plating (SEP), which deposits silver nanoparticles exclusively where these moieties exist, i.e., on the surfaces of the structures, transforming them into conductive ones, as described in detail in refs (28,59).

The MPP fabrication of the structure was done using a hybrid photoresist with Zr-based inorganic component^60^ (see SI). The photoresist shows dielectric properties, and a postmetallization process was required to obtain a conductive structures suitable for the low THz regime. After a carefully designed SEP protocol (see SI) based on previous work that has been done for various applications, we achieved a silver nanoparticle coating of final thickness, overcoming the skin depth of silver in low THz frequencies.

**Figure 7:**
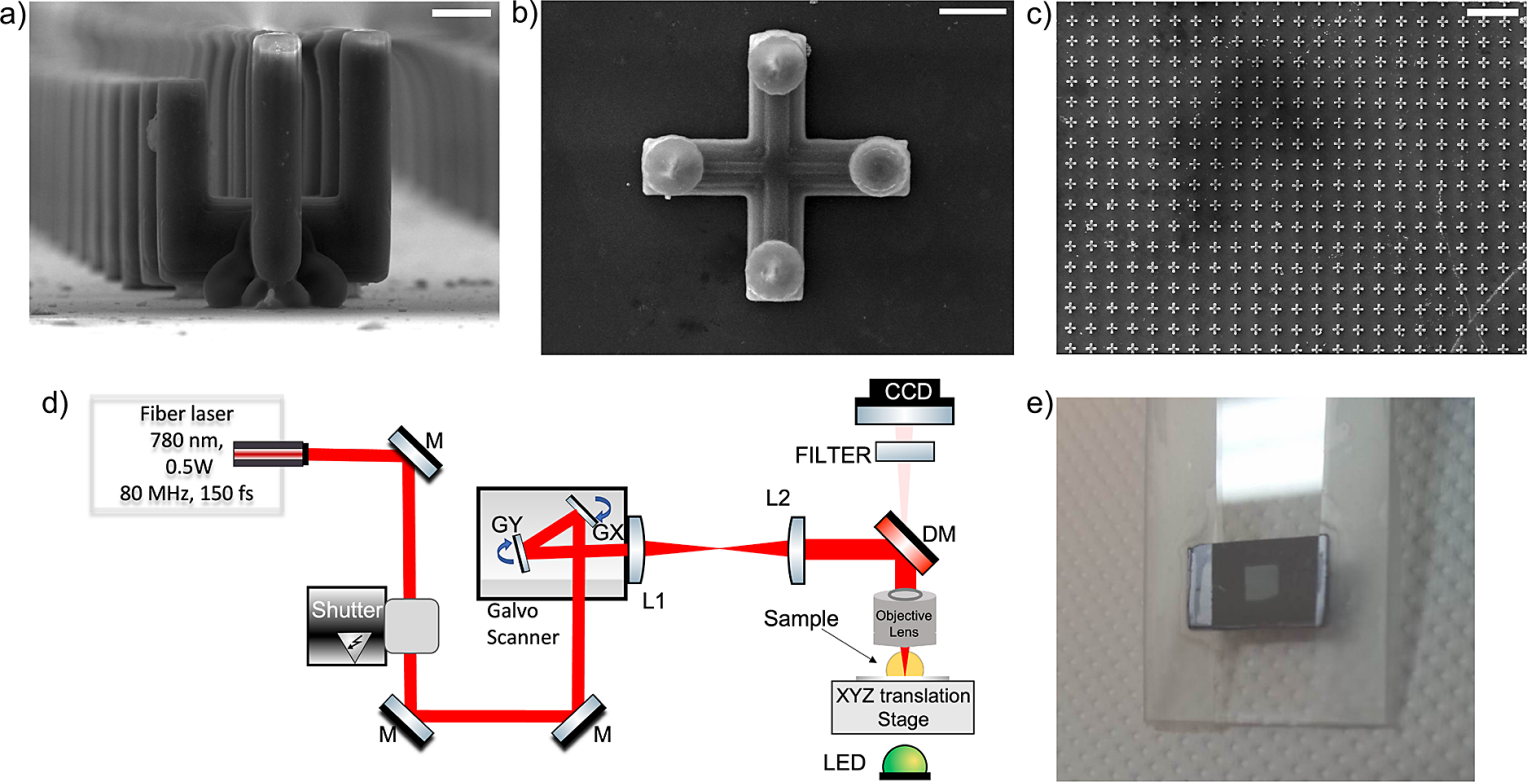
SEM images of the fabricated metamaterial in (a) side view (unit cell), (b) top view (unit cell), and (c) top view (array). (d) Schematic presentation of the homemade multiphoton polymerization setup. M; silver-coated mirrors, L1; F-theta lens, L2; scan lens, DM; dichroic mirror; GX & GY; galvanometric mirrors. (e) Photograph of the metamaterial device fabricated on the silicon substrate. Scale bar is (a,b) 10 μm and (c) 200 μm.

The sample was processed using the optical setup depicted in Figure 7d. The setup includes a femtosecond fiber laser (FemtoFiber pro NIR, Toptica Photonics AG) emitting at a central wavelength of 780 nm, with pulse duration of 150 fs, an average output power of 500 mW, and a repetition rate of 80 MHz, a 2D Galvo scanner system (Scanlabs HurryscanII 10) consisting of galvanometric mirrors that scans the laser beam on the *xy* plane during the fabrication process, an acousto-optical shutter, and a high-resolution *xyz* axis system. A microscope objective lens with 40× magnification (Zeiss, Plan Apochromat) with numerical aperture 0.95 was employed to focus the laser beam onto the photoresist.

Regarding the dimensions of the fabricated structures, the unit cell size was 80 × 80 μm^2^ in the *xy* plane. The entire processed area comprises approximately 1700 unit cells and occupies ∼3.4 × 3.4 mm^2^ (Figure 7e) in order to overcome the THz beam diameter in the characterization process which is 2 mm. The manufacturing of this area required approximately 7 h. The resulting 3D U-shaped resonators are depicted in Figure 7, which shows SEM images with different magnifications and orientations of the structure. All SEM images distinctly illustrate the high stability and uniformity of the structures (Figure 7a,c), key characteristics for achieving reliable functionality in a metamaterial. The deformed bottom bases in single meta-atoms, which arose due to the reflective properties of silicon against 780 nm laser radiation enhancing the polymerization of the material close to the substrate, are minor structural imperfections insignificant for the working principle of the proposed metamaterial. This is because all meta-atoms exhibit the same defect, and this defect does not affect the electromagnetic structure response. Note that the EIT mechanism relies on the asymmetry of one U-shape resonator, where one arm is shorter than the other one, as shown in Figure 7a; this feature was perfectly processed.

**Figure 8:**
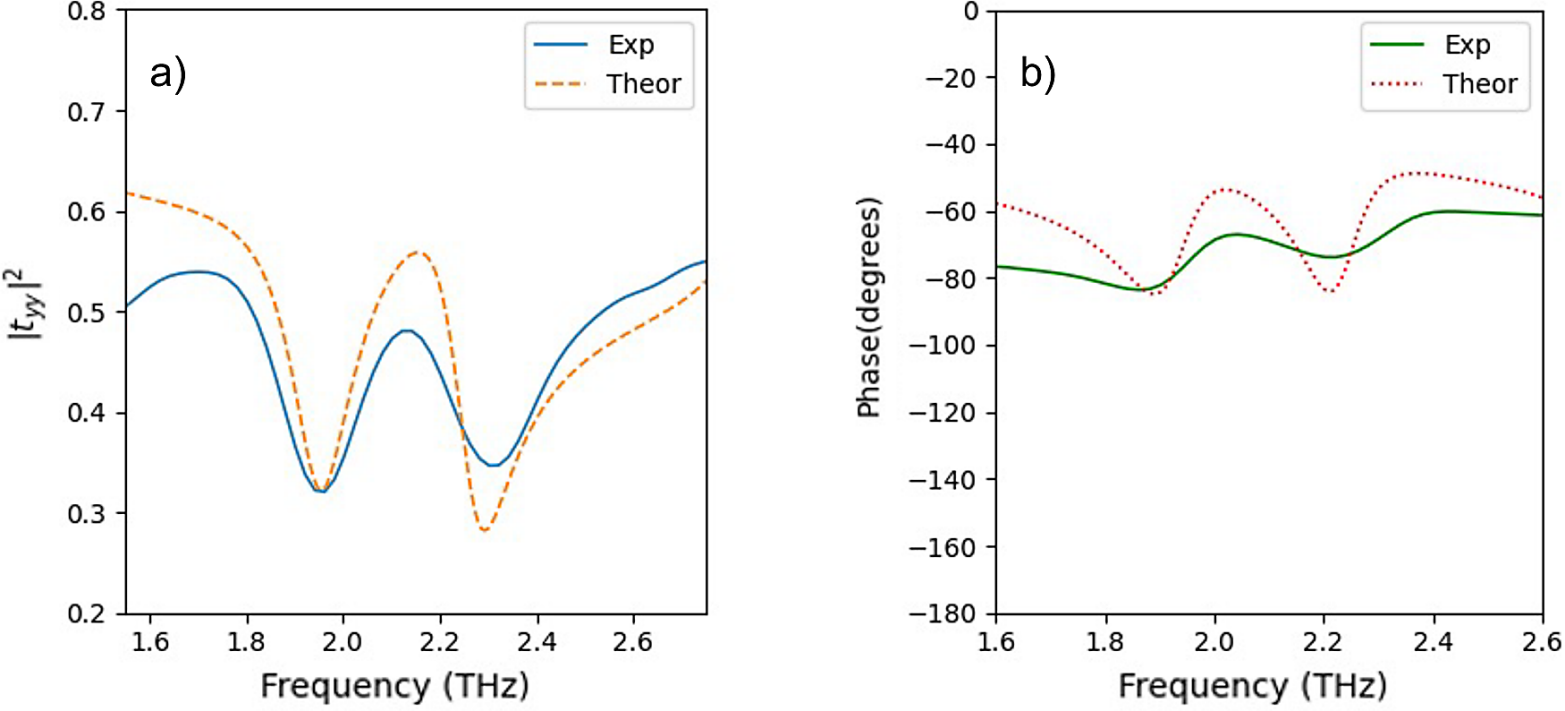
(a) Transmission (power) coefficient for linearly polarized light along the *y* axis (*T*_*yy*_ = |*t*_*yy*_|^2^). (b) Corresponding transmission phase. Blue curves show the experimental data; red curves show the corresponding theoretical ones.

In order to study the THz response of the fabricated sample, a THz-TDS system^61^ based on photoconductive antennas (TOPTICA TeraFlash pro) and operating in transmission mode is employed.^28^ The transmission through the sample THz electric field is recorded by the detector placed behind the silicon substrate. By rotating the sample, we were able to detect the linearly polarized wave along the *y* axis and measure the transmission coefficient *T*_*yy*_. This transmission, along with the corresponding theoretical results, is shown in Figure 8.

For the theoretical result, in order to get the optimum fitting to the experiment, the calculations were done with the dimensions and the parameters of the fabricated structures. From the SEM images in Figure 7, the dimensions were measured to be *d* = 8.9 μm, *L* = 40 μm, α = 79.8 μm the size of the unit cell (lattice constant), *h* = 35.2 μm, and *c* = 8.1 μm. The silver nanoparticles that coat the structure were measured to be approximately 150 nm in size by using SEM images with high magnification (see SI). This difference in dimensions of the structure as well as the thinner silver coating of the polymer creates a shift in the resonances of the EIT to higher frequencies, as it was predicted also from the parametric study of different pillar heights (see SI). In addition, the final simulations take into account the final sample properties. The silicon substrate with relative permittivity of ϵ_*s*_ = 11.2 and loss tangent tan δ = 0.02 is included in the calculations. The conductivity of the silver coating is set to be σ_exp_ = 5.75 × 10^5^ S/m, as found by conductivity measurements (see SM); this value is lower than the one that has been measured previously in the literature.^62^ The lower conductivity is attributed to multiple repeats of the metallization process in order to reach the desired thickness. This generates a multilayer system of silver nanoparticles, which decrease the mobility of the carriers on the surface^63^ (similar to the single-layer and multilayer graphene system^64^). In addition, the surface roughness (which may be a few nanometers) can effectively lead to lower conductivity through scattering effects.^65^

Lower conductivity results in reduced transmission of the THz radiation through the sample, as theoretical parametric simulations for different silver conductivity values have shown (SI). Thus, the intensity of the transmission was expected to be relatively low. However, the EIT phenomenon was still observable and not affected by the conductivity of the metallic structure, since it is triggered by the broken symmetry imported in the system and not significantly by its electric properties.

From the TDS characterization, we obtained an electric signal in time, and using Fourier transformation, we get the amplitude/phase in the frequency domain. Dividing the detected signal of the bare silicon substrate with the manufactured one, we obtain the normalized transmission spectra for the proposed metamaterial (SI). As mentioned already, the experimental results for transmission, as well as the theoretical fitting from the simulations, are presented in Figure 8a. From the experimental curve (blue curve), we observe that despite the increased losses, the characteristic EIT spectral feature is clearly visible. This is verified by the theoretical calculations (orange curve) incorporating the experimental conditions, which show good agreement with the experimental data. In addition, the EIT nature of the response is also corroborated by the transmission phase, as depicted in Figure 8b.

The behavior of the EIT response from the experiment comes in alignment with the theoretical predictions from the aspect of where the resonances are expected to be, with the exact dimensions of the fabricated structure and the experimental conditions included in the simulations. In the experimental part, we observe the transmission peak of EIT at *f* = 2.13 THz. The narrow EIT window (a bandwidth of *f* = 0.43 THz with high transmission between two transmission dips) shows promising results for delaying light applications. In addition, the transmission phase was measured again using TDS, and the final results for the transmission phase, which triggers also the delaying light applications as it was described in subsection “EIT response,” are exhibited in Figure 8b.

The enhanced losses observed in the characterization of the structure can be explained by the metallization process, as it was described above, the substrate’s resistivity, and the non-negligible amount of dirt and silver nanoparticles on the substrate, which are responsible for the absorption of a small part of the THz radiation before it reaches the detector. In addition, the silver nanoparticles are not formed completely homogeneously onto the polymerized material.

## Discussion

In this paper, we demonstrated the design and fabrication of a metallic metamaterial exhibiting EIT associated with enhanced RI sensing performance in low THz frequencies. Our design resembles a cactus, featuring two vertical metallic U-shaped rings arranged perpendicular to each other. One of the rings has broken symmetry, crucial for inducing a coupling between a dark and a bright resonance, leading to the EIT feature. The structure was realized experimentally by using MPP and selective electroless silver plating. Its electromagnetic response was experimentally measured through THz-TDS and has been verified against numerical simulations, taking into account the actual dimensions and realistic material parameters. In the theoretically optimized metal-coated structure, we achieved a transmission amplitude of 80% at the EIT peak, along with a group delay of 1.3 ns (∼2200 carrier cycles). The investigation of the RI sensing performance of the structure yielded a quite high FOM (≈34). This advanced sensing potential originates from the sharp EIT feature combined with the 3D meta-atom geometry associated with a large surface area for the structure-analyte interaction. The proposed structure and research underscore the potential of 3D-meta-atom configurations for advanced applications in slowing light and environmental sensing, offering insights also into the design and fabrication challenges associated with 3D meta-atom-based metamaterials.

## Materials and Methods

A suitable photoresist for processing via MPP and subsequent postprocessing via SEP is a metal-binding photopolymer to which 2-(dimethylamino) ethyl methacrylate is added at a concentration of 30% v/v relative to the base ZPO monomers.^60,66^ In this study, the modified photoresist was synthesized in-house (see SI for details) and then drop-casted onto high-resistivity silicon substrates (thickness: 540 μm, resistivity: 100–1000 Ω·cm) that exhibit semitransparent optical properties in the THz region of interest. Prior to deposition, the silicon substrates were silanized to enhance adhesion between the processed structures and the silicon surface. For better attachment of structures on the substrate, a monolayer of 3- (t*rimethoxysilyl*)*propylmethacrylate* (MAPTMS) was formed on the surface of the substrate following a silanization process. The substrates were immersed in a solution of ammonium hydroxide (*NH*_4_*OH*) and hydrogen peroxide (*H*_2_*O*_2_) at a volume ratio 3:1 and heated at 75^o^ for 15 min in order to clean the surface. Subsequently, they were immersed in distilled water and dried. Finally, the silanization of the substrates was completed by immersing them in a solution of toluene and MAPTMS at 0.5% *v*/*v* and leaving them overnight. Next, the substrates were cleaned using ethanol or acetone and stored in ethanol in a cool and dark environment. 40 μL of the photosensitive material was drop-casted using a pipette. The drop-casted material was kept under low vacuum conditions at room temperature for 2 days, ensuring the complete evaporation of any residual solvents. After the fabrication process, the sample was immersed for 45 min in 4*methyl-*2*pentanone* and then rinsed in *isopropanol* for another 30 min. Here, in order to achieve the selectivity of SEP, it is important to remove all excess nonpolymerized material from the sample and the substrate in order to avoid the deposition of silver nanoparticles onto them.

The fabrication of single 3D U-shaped resonators was carried out in a layer-by-layer approach, following a bottom-to-top strategy, using a slicing and hatching distance of 1 μm and 500 nm accordingly. More specifically, the process started with the fabrication of the taller pillars, followed by the arms, and finishing at the bottom base of the structure using a galvo scanning velocity of 3 mm/s and an average laser power of 100 mW, as measured by a digital power meter positioned in front of the last mirror before the galvo scanner system.

The metallization process that was used is a modification of the one established in ref (62) and further demonstrated in refs (28 and 67). SEP is a straightforward chemical procedure that does not require any application of electrical potential and provides silver nanoparticle coating in a highly selective way only on the surface of the polymerized material. In THz frequencies, the skin depth of silver is approximately 80 nm,^68^ and thus the protocol was modified to fulfill this condition. From the SEM images depicted in the Supporting Information, the formed coating on the surface of the polymerized material was roughly 150 nm. Since the coating is not completely homogeneously placed, the thickness can only be estimated using the mean value of the size of silver nanoparticles that are formed on the surface. Energy-dispersive X-ray spectroscopy experiments were also done in order to observe the presence of silver on the fabricated structure.

## Supporting Information Available

Setup of simulations, parametric studies of critical parameters of the metamaterial, multipole analysis, circuit model describing the EIT metamaterial, further experimental information regarding fabrication setup, photopolymer, and silver electroless plating (PDF)
