# Supplementary material for: Cactus-like Metamaterial Structures for Electromagnetically Induced Transparency at THz frequencies
Source: ACS Photonics. 2024 Oct 7;12(1):87–97. doi: 10.1021/acsphotonics.4c01179 (PMC11748748; doi:10.1021/acsphotonics.4c01179)
Supplement: Supplementary file 1 — ph4c01179_si_001.pdf [file ph4c01179_si_001.pdf]

Supporting Information: Cactus-like Metamaterial Structures for Electromagnetically Induced Transparency at THz frequencies

Savvas Papamakarios,[*,†,‡] Odysseas Tsilipakos,[¶] Ioannis Katsantonis,[†] Anastasios D. Koulouklidis,[§] Maria Manousidaki,[†] Gordon Zyla,[†] Christina Daskalaki,[†] Stelios Tzortzakis,[†,∥] Maria Kafesaki,[*,†,∥] and Maria Farsari[*,†]

†*Institute of Electronic Structure and Laser, Foundation for Research and Technology - Hellas (FORTH-IESL), GR-70013 Heraklion, Crete, Greece*

‡*Department of Physics, National and Kapodistrian University of Athens, GR-15784 Athens, Greece*

¶*Theoretical and Physical Chemistry Institute,National Hellenic Research Foundation, GR-11635 Athens, Greece*

§*Department of Physics and Regensburg Center for Ultrafast Nanoscopy (RUN), University of Regensburg, 93040 Regensburg, Germany*

∥*Department of Materials Science and Engineering, University of Crete, GR-70013 Heraklion, Crete, Greece*

E-mail: spapamakarios@iesl.forth.gr; kafesaki@iesl.forth.gr; mfarsari@iesl.forth.gr

This Supporting Information document is 24 pages long and includes 16 figures. It is structured in 10 sections:

S1. Setup of the simulations

S2. Parametric studies

S3. Multipole analysis and further parametric study

S4. Circuit model describing the EIT metamaterial

S5. Photosensitive material

S6. Sample preparation (Si substrate)

S7. Multiphoton Polymerization (MPP)

S8. Characterization

S9. Silver Electroless Plating

S10. Conductivity measurement

S 1 Setup of the simulations

Numerical calculations were carried out with the commercial software packages CST Microwave Studio and COMSOL Multiphysics, employing the finite element method in the frequency domain. A fine size mesh of tetrahedra was chosen according to the "physics-controlled mesh" option in COMSOL and the "adaptive mesh refinement" option was selected in CST. The transmission and reflection coefficients were simulated for one unit cell with periodic boundary conditions on the x- and y- boundaries (see Figure 1 of the main text). The incident wave was introduced from the top and x- or y-linearly polarized waves were injected in the simulation domain.

S 2 Parametric studies

In Figure S1 we present three different parametric studies of the "cactus-like" structure, in order to fully demonstrate the electromagnetic response for different parameters that may affect the EIT effect. First, in Figure S1(a) the symmetric vertical-arms of the structure (parameter h) are varying while the cut is fixed at $c = 8\ \mu m$. Therefore, the structure is

becoming shorter. The result, for y-polarized illumination, is that the EIT phenomenon shows a frequency shift, highlighting the spectral tunability that this 3D structure offers as a degree of freedom. Next, in Figure S1(b), the case of illuminating the sample with linearly polarized light along x axis is presented, where the magnetic field now is perpendicular to the symmetric ring. In this case, the varying value is the cut c, while the symmetric pillars remain at the value of $h = 40\ \mu m$. In this case the transmission response does not change at all, showing that depending on the orientation of the sample in the $x - y$ plane, EIT phenomenon with the excitation of dark and bright resonances can occur or not. In fact, in the case of x-polarized incident wave the resonance excited is the magnetic resonance of the symmetric U-ring, without any coupling to the arms of the asymmetric, perpendicular ring. That's why the change in the asymmetric ring does not affect the structure response. Finally, in Figure S1(c) the variable is the unit cell size, a, of the meta-atom. In this case, the coupling between near-neighbouring meta-atoms is affected. The transmission response is not affected significantly, except for $a = 80\ \mu m$ where the unit cell size exceeds the wavelength of the excitation and we get higher diffraction orders in the specific frequency spectrum that we simulate.

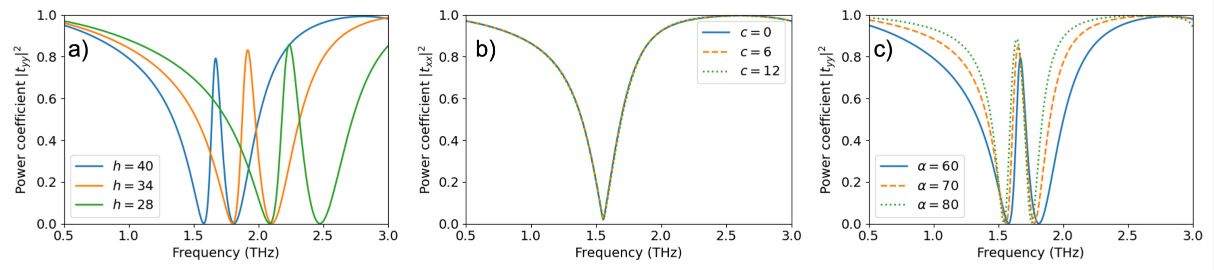

Figure S1: Parametric study of the proposed unit cell, showing the transmission coefficient for (a) different values of h (symmetric feature of cactus-like design, see Figure 1 of main text); T_{yy} is shown; (b) different values of the cut c and incident polarization along x-axis; T_{xx} is shown, (c) different unit cell sizes, a, for y-polarization (T_{yy} is shown). All size parameters in the labels are in μm.

In addition, two critical parameters that affects the results of our study are the cut parameter c and the conductivity of the silver that was used. As it was discussed in the main text, the size of the asymmetry results in different values of group delay as the shorter

pillar in the metamaterial is responsible for the change in group delay. As it is presented in Figure S2 (a) as c increases it reach a maximum value of 1.3 ns in a narrow bandwidth and then it starts to decrease due to the broader EIT peak which simultaneously creates a broader bandwidth.

A significant parameter also that has to be taken into account is the different values of conductivity that are achieved using SEP (see S9 in detail) compared to the theoretical ones that were used in the calculations. In Figure S2(b) the parametric study for different values of conductivity σ is presented. It is clear that lower conductivity has a significant impact on the transmission amplitude as the EIT peak is getting lower and the EIT window is narrower. This result is related to the fabricated sample which has been metalized in order to compare the experimental result of transmission with the theoretical one.

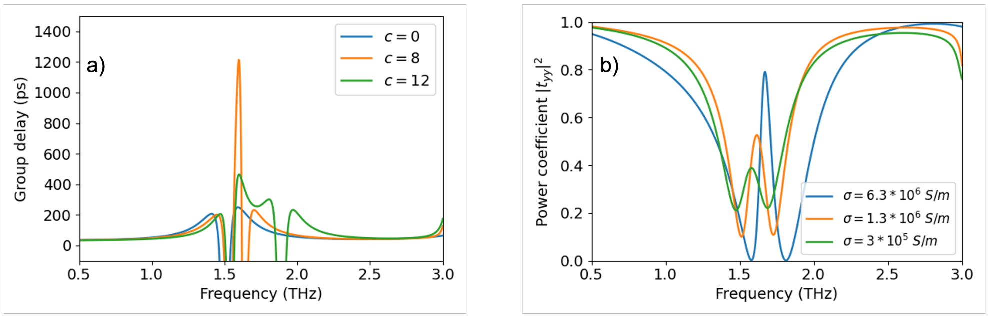

Figure S2: (a) Parametric study of the proposed unit cell, showing the group delay performance for different values of the cut parameter c (in μm) and (b) transmission coefficient T_{yy} for different values of silver's conductivity σ.

Finally, in order to exploit the broad detecting range of the proposed design, we study the sensing performance for analytes in the RI range of $n = 1.5 - 1.8$. (In the main text, RIs in the range 1-1.4 were covered.) The corresponding reflection, transmission, and absorption coefficients are depicted in Figure S3(a), (b), and (c), respectively, for the case $c = 4\ \mu m$. The proposed meta-atom can detect the change in analyte refractive index with a sensitivity to be $S = 0.825$ THz/RIU. This value is slightly lower than the one for calculated for refractive indices in the range $n = 1 - 1.4$.

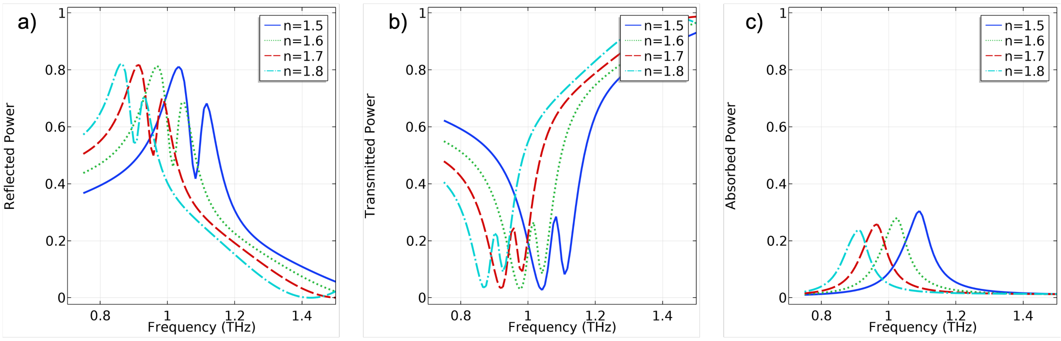

Figure S3: Sensing performance of the proposed metamaterial for analytes with refractive index in the range 1.5-1.8. (a) Reflected power, (b) transmitted power, and (c) absorbed power.

S 3 Multipole analysis and further parametric study

To analyze and better understand the different resonant modes involved in our structure, we have used the induced current in the plane wave scattering simulations to perform a multipole analysis. In Figure S4, we present transmission and reflection coefficients for plane-wave scattering, for a y-polarized incident wave, for cut sizes $c = 0$ and $c = 8$. In both cases, we then perform a multipole expansion of the induced conduction current in order to distinguish between scattering by the different terms (moments) of the decomposition. Finally, we used the moments to reconstruct the reflection/transmission coefficients; this reconstruction is presented also in Figure 2, showing excellent agreement with the plane wave scattering results, demonstrating the accuracy and adequacy of the expansion (i.e. no need to include further terms).

More specifically, in Figure S4(a) we depict the reflection and transmission power coefficient for the symmetric metamaterial structure (no cut) (both by scattering simulations and multipole reconstruction) and in Figure S4(b) the corresponding multipole decomposition. On the resonance frequency it is the p_y and m_x contributions peaking, as shown also in the main text. The resonance that is excited is the magnetic resonance of the parallel to the incident electric field U-ring, which is coupled both to the electric and magnetic incident field components.

The corresponding data for the asymmetric structure ($c = 8$ μm) are shown in panels (c) and (d). The quasi-dark mode ($f = 1.758$ THz) can be now excited and interferes with the bright mode since they lie close in frequency. The interference between the two modes can be seen in the multipole expansion as well. Since the two modes have similar multipole composition, the excitation of the quasi-dark means that the respective p_y and m_x contributions will interfere. This is evident in the dips of the p_y and m_x contributions near $f = 1.758$ THz.

Exploring in detail the electric and magnetic field profile and the currents at the EIT peak, we see that each vertical arm of the cactus-structure exhibits a magnetic resonance (antiparallel currents) with its two nearest neighbors, i.e. the structure behaves as four magnetic resonators at the four vertical sides of the cactus. Due to the mirror symmetry of the structure in the x-direction the total induced dipoles p_x, m_y sum up to zero, which is not the case in the y-direction and the associated dipoles p_y and m_x.

We have further investigated the robustness of the EIT effect with c (cut parameter) varying from 0 to 10 μm for a normally-incident y-polarized [Figures 3(a)-(b)] and x-polarized [Figures 3(c)-(d)] plane wave. In the first case, the EIT peak appears and gradually becomes stronger and broader as c varies from 2 μm to 10 μm. On the other hand, in the second case a single reflection band (corresponding to the bright mode) is observed throughout.

The impact of c on the far-field contributions from each multipole moment are depicted in Figure 4. We find that the contributions of the electric dipole (p_y) and magnetic dipole (m_x) moments significantly increase with c, leading to stronger radiative damping (i.e., free-space coupling) for the quasi-dark mode. The corresponding Q-factors are also presented in the table 1. We observe that the initially dark mode (Q_{rad} infinite) becomes quasi-dark when $c \neq 0$ with Q_{rad} steeply decreasing as c increases. Note that to calculate Q_{rad} we momentarily zero-out resistive losses in the system, in which case Q_{tot} reduces to Q_{rad}.[1] Q_{res}, which is associated with ohmic losses, is calculated by making use of ${Q_{tot}}^{-1} = {Q_{rad}}^{-1} + {Q_{res}}^{-1}$.

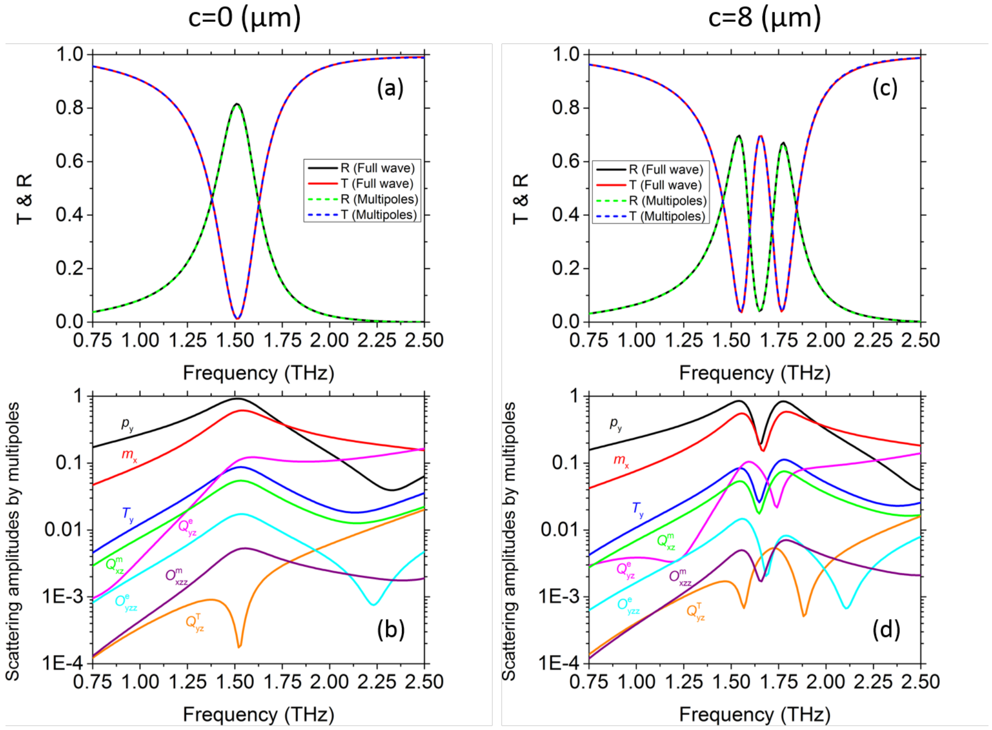

Figure S4: (a) Reflected and transmitted power of the symmetric metamaterial structure ($c = 0\ \mu m$) calculated by the full wave simulations and by the summation of the contributions of the eight multipole moments shown in (b). (b) Scattering amplitude contributions by the individual multipole moments of the symmetric metamaterial structure. (c) The same as (a) for the asymmetric structure with $c = 8\ \mu m$. (d) The same as (b) for the asymmetric structure with $c = 8\ \mu m$.

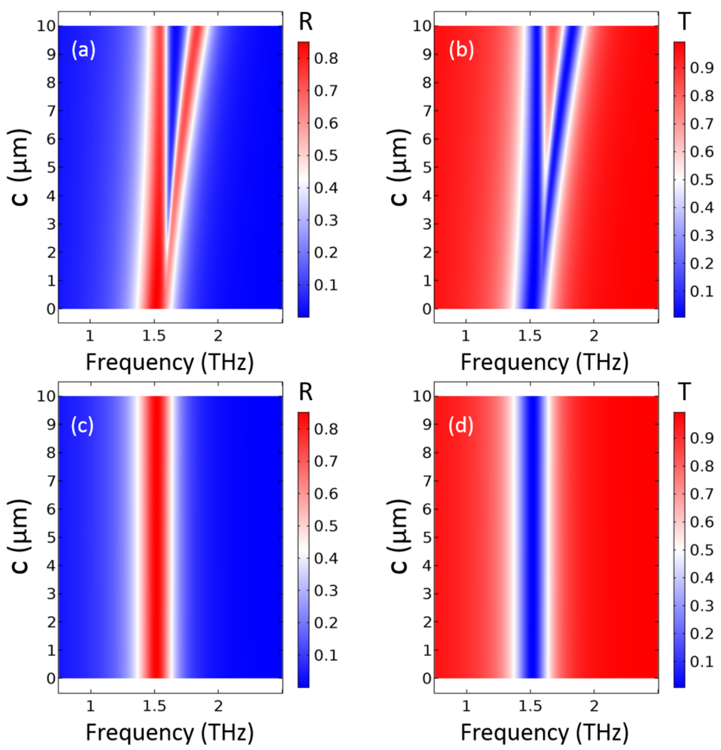

Figure S5: Reflection and transmission spectra as a function of c (cut parameter) (a)-(b) for wave polarized along y-axis and (c)-(d) for wave polarized along x-axis.

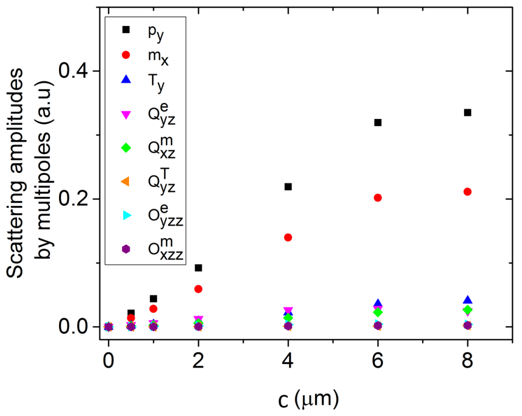

Figure S6: Evolution of multipole contributions to the scattered field for the (quasi-) dark mode as the cut-parameter c varies. When $c = 0$, p_y and m_x are zero and the mode is dark.

Table 1: Resonant frequency and quality factors for the (quasi-)dark mode as the cut-parameter c varies.

c (μm)	f (THz)	Q_{rad}	Q_{res}	Q_{tot}
0	1.589	Inf ($> 10^7$)	55.56	55.56
0.5	1.591	3210	54.56	53.65
1	1.596	765.75	51.8	48.52
2	1.606	175.67	42.15	33.99
4	1.628	31.34	19.4	11.98
6	1.694	15.31	12.3	6.82
8	1.758	13.93	11.43	6.28

S 4 Circuit model describing the EIT metamaterial

Having shown the equivalence of the circuit equations Eqs. (1), (2) in the main text to a dark-bright coupled resonator system, we can proceed calculating the electromagnetic response (polarizabilities and through them scattering coefficients) of our system. The electric and magnetic dipole moments produced by the current I_A (the only one that radiates) are given (approximately) by $p_y = Q_A d = iI_A d/\omega$ (Q_A denotes charge) and $m_x = I_A A$. The current I_A, calculated by solving Eqs. (1) and (2) in the main text, can be expressed as

$$I_A = -\frac{\Delta_0 - \omega^2(M/L)}{L[\Delta_1\Delta_2 - \omega^4(M/L)^2]}(i\omega d E_y + \mu_0\omega^2 A H_x), \tag{1}$$

where $\Delta_0 = \omega^2 - \omega_0^2 + i\omega(R_0/L)$, $\Delta_1 = \omega^2 - \omega_1^2 + i\omega(R/L)$, $\Delta_2 = \omega^2 - \omega_2^2 + i\omega(R'/L)$ and $\omega_1^2 = 1/LC$, $\omega_2^2 = 1/LC'$.

Having the current we can explicitly write the electric and magnetic dipole moments p_y and m_x, and through them obtain approximate formulas for the structure polarizabilities, defined by[2,3]

$$\mathbf{p} = \bar{\bar{\alpha}}_{ee}\mathbf{E} + \bar{\bar{\alpha}}_{em}\mathbf{H}, \quad \mathbf{m} = \bar{\bar{\alpha}}_{mm}\mathbf{H} + \bar{\bar{\alpha}}_{me}\mathbf{E}. \tag{2}$$

In equations (2) $\bar{\bar{\alpha}}_{ee}$, $\bar{\bar{\alpha}}_{mm}$, $\bar{\bar{\alpha}}_{em}$, $\bar{\bar{\alpha}}_{me}$ are averaged (effective) electric, magnetic, electromagnetic, and magnetoelectric polarizability tensors of the double-U structure and the $\mathbf{E}, \mathbf{H}$ indicate applied fields (we omit for simplicity any coupling between unit cells). In our case

Eqs. (2) give $p_y = \alpha_{ee}^{yy} E_y + \alpha_{em}^{yx} H_x$, $m_x = \alpha_{me}^{xy} E_y + \alpha_{mm}^{xx} H_x$.

Relating p_y and m_x with the current I_A as mentioned above, the electric, α_{ee}^{yy}, and magnetic, α_{mm}^{xx}, polarizabilities of our structure (the only ones that contribute to the transmission and reflection) can be written as

$$\alpha_{ee}^{yy} = \frac{d^2}{L} \frac{\Delta_0 - \omega^2 (M/L)}{\Delta_1 \Delta_2 - \omega^4 (M/L)^2}, \tag{3}$$

$$\alpha_{mm}^{xx} = -\frac{\omega^2 \mu_0 A^2}{L} \frac{\Delta_0 - \omega^2 (M/L)}{\Delta_1 \Delta_2 - \omega^4 (M/L)^2}. \tag{4}$$

(Note that for identical rings (with no cut) $\Delta_1 = \Delta_2 = \Delta_0$, and the above expressions give the polarizabilities of a single U-ring.) The reflection and transmission coefficients of our structure, which are crucial for the understanding of its electromagnetic response, can be obtained via the general relations,[2] $t = 1 + \frac{i\omega Z_0}{2A_{uc}}[\alpha_{ee}^{yy} + (\mu_0/Z_0^2)\alpha_{mm}^{xx}]$ and $r = \frac{i\omega Z_0}{2A_{uc}}[\alpha_{ee}^{yy} - (\mu_0/Z_0^2)\alpha_{mm}^{xx}]$, where Z_0 is the wave impedance in free space and A_{uc} the area of the metamaterial unit cell.

S 5 Photosensitive material

The 3D printable hybrid material that is used in this paper is a modified version of SZ2080^{TM}.[4] Basic components of the material are zirconium (Zr) and silicon (Si).[5] For the organic part 3-(Trimethoxysilyl) propyl methacrylate (MAPTMS) and 2- (Dimethylamino)ethyl methacrylate (DMAEMA) are used, where the DMAEMA component is the organic photopolymerizable monomer. For the inorganic part Zirconium n-propoxide (ZPO) was used. In combination with the alkoxysilane groups of MAPTMS, that can undergo hydrolysis and condensation, served as the inorganic network forming moieties. ZPO is used to provide mechanical stability to the polymerized material. In the present research the photoinitiator (PI) 4,4′-Bis(diethylamino) benzophenone (Bis)[6] is used, which absorbs strongly at 400nm but weaker at 800nm.[7] This state is a prerequisite for a functional PI, as it has to be transparent at the laser's central wavelength (780 nm) and simultaneously to have an absorption peak at the two-photon absorption wavelength ($\lambda/2$). Bis makes MPP more efficient in lower energy values and does not affect at all the organic-inorganic network of the hybrid materials. Synthesis of the hybrid material is a complex process where a variety of reactions take place, such as condensation and hydrolysis.[5] First, MAPTMS and 0.1M HCL (diluted in water) at 10:1 ratio are combined in a flask and stirred for 15 minutes until the solution become homogeneous and completely transparent. Hydrolysis takes place in the solution immediately and after the stirring the hydrolyisis of the alkoxysilane groups is completed. The second solution is ZPO with DMAEMA. ZPO is highly reactive with moisture so it is diluted in 70% w/w concentration of propanol. DMAEMA is slowly added to ZPO at a molar ratio 7:3, where DMAEMA serves as a carrier for ZPO and no reaction takes place. DMAEMA is responsible for the polymeric characteristic of the material and provides also metal-binding moieties that are crucial for the metallization of the material with a post-treatment electroless coating technique. Again, the solution is stirred for 15 minutes to become homogeneous. After the two components have homogeneously disolved, the partially hydrolyzed MAPTMS

is slowly added to the zirconate complex in a droplet-by-droplet approach. In this case, the organic-inorganic network is formed due to the Si atoms that are attached to the polymer matrix. The solution is stirred for 15 more minutes until it becomes transparent and consistent. Finally, the photoinitiator, Bis, is added to the final solution at 1% w/w concentration. The final solution is stirred for 15 minutes until all of the components are disolved. Afterwards, the final solution is filtered using 0.45 μm syringe filters[8] . The material was synthesized following the detailed protocol in ref.[4] The material then is sealed and stored in a cool environment (fridge) to protect it from heat, light and moisture and make it last longer, since its basic components are highly sensitive to environmental stimuli. DMAEMA must be stored in fridge also.

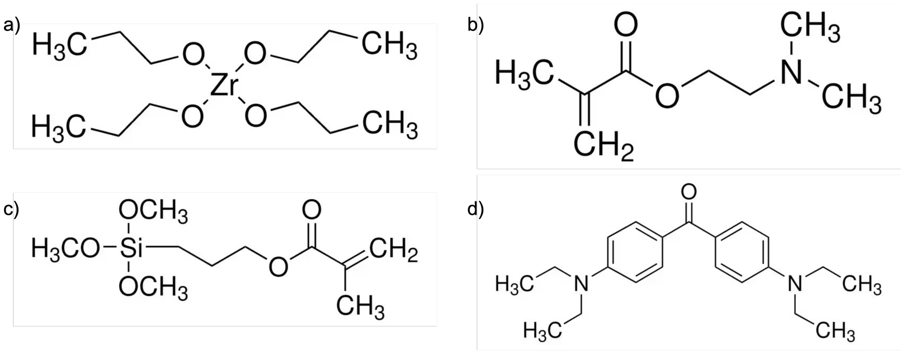

Figure S7: Chemical structural formula of basic components of the metal-binding photopolymer that was used in this work. (a) ZPO, (b) DMAEMA, (c) MAPTMS, (d) BIS .

S 6 Sample preparation (Si substrate)

High resistivity Silicon substrates ($\sim 1\times1$ cm^2, 540 μm thickness) were used for the purpose of the present research. These specific substrates show a resistivity of $100-1000$ Ω·m and are usual electromagnetic conductors that exhibits transmitting properties at the frequencies of interest ($1-4$ THz) for the purpose of our work, as it was measured and shown in Figure S8. In order to create strong bonding conditions between the polymerized photoresist and the substrate, a monolayer of 3-(Trimethoxysilyl)propyl methacrylate (MAPTMS) has to be formed on the surface of the substrate following a silanization process. The substrates are immersed in a solution of Ammonium hydroxide (NH4OH) and Hydrogen peroxide (H_2O_2) at a volume ratio 3 : 1 and heated at 75 ^oC for 15 minutes in order to clean the surface. After, they are immersed in distilled water and dried. Finally, the sinalization of the substrates is completed by immersing them in a solution of Toluene and MAPTMS at 0.5% v/v and let them rinsed for 24 hours. Finally, the substrates are cleaned using ethanol or acetone and stored in ethanol in cool and dark environment. Transmission spectra of the Si substrate that was used for the targeting frequencies is shown in S8, where it is obvious that the substrate turns slightly transparent in these frequencies. Substrates have to be transparent in the region of interest so transmission measurements can be taken. For the preparation of the sample, the substrate is dried using nitrogen gas stream. A volume of the photosensitive material are placed on the substrate using drop casting technique with a pipette. Then the sample must be placed on a hot plate for at least 1 hour at 60 ^oC, or for at least 24 hours under low vacuum conditions in room temperature. This has a result the formation of the sol-gel material that the excessive solvents have been evaporated. Then, the sample is ready for use. After the fabrication process, the sample is immersed for 30 minutes in 4-methyl-2-pentanone and the rinsed in 2-propanol for another 30 minutes. Here it is important to remove all the excess non-polymerized material from the sample in order to avoid the metallization of extensive material which will lead to losses during the characterization.

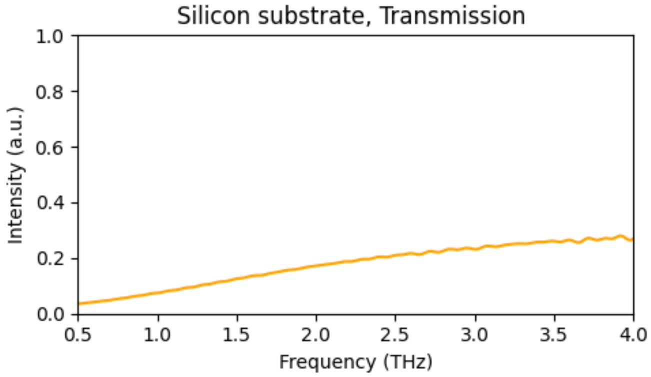

Figure S8: Measured transmission of high-resistivity Si substrate.

S 7 Multiphoton Polymerization (MPP)

MPP is a true 3D printing process relying on Direct Laser Writing,[9] suitable for optical, mechanical, imaging and photonic applications with great capability on printing 3D arbitrary structures reaching to a resolution of sub microns.[10] Relying on non-linear optics, MPP uses the two photon (or more) absorption as a triggering mechanism in a very small volume of material, significantly smaller that other techniques, to initialize the polymerization process. On the benefits are the maskless production of the samples, in contrast with conventional lithographic printing techniques, using low cost photosensitive material and the fabrication speed.[11] Varying the components of the photosensitive material can provide different chemical, optical and mechanical properties of the fabricated structure resulting in different applications. A femtosecond laser beam is tightly focused within a focal volume of the photoresist, triggering the two-photon absorption which initialize the chain reaction of the solidification of the resist fabricating the structure with a layer-by-layer slicing process.[12] The main components of the setup for the fabrication of the proposed metamaterial structure are consisting of the irradiation source for the MPP process which is a femtosecond fiber laser (FemtoFiber pro NIR, Toptica Photonics AG) emitting at 780 nm , with pulse duration of 150 fs, average output power of 500 mW and repetition rate of 80 MHz. Then, the beam is inserted in a galvo scanner system (Scanlabs HurryscanII 10) that scans the

laser beam on x-y plane during the fabrication process. The laser bear was focused using a 40x microscope objective lens (Zeiss, Plan Apochromat) with N.A.=0.95 was used. The sample was placed on xyz linear translation stages (Physik Instrumente) which are suitable for fabrication of the design using layer-by-layer method with high precision on z axis, while the x and y stages are mandatory for the fabrication of large areas using stitching technique with high precision as well. In Figure S9, L_1 refers to a telecentric lens and L_2 scan lens.

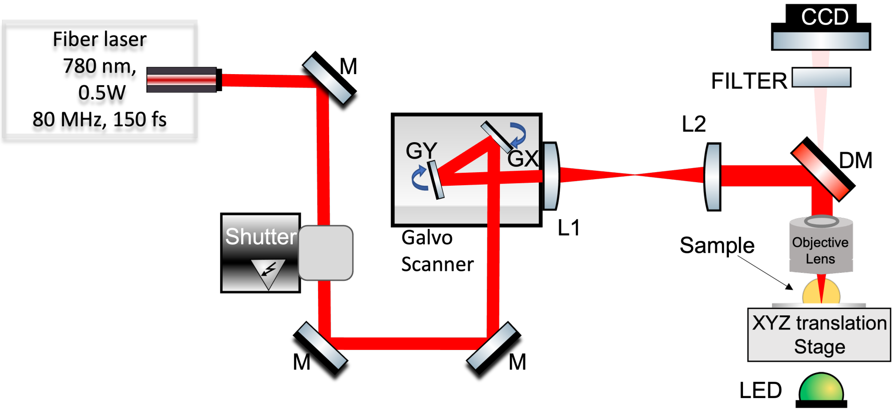

Figure S9: Schematic presentation of homemade multiphoton polymerization setup. M; silver-coated mirrors, L1; F-theta lens, L2; scan lens, DM; dichroic mirror, GX & GY Galvanometric mirrors.

For the proposed metamaterial, the fabrication begun from the bottom (Si substrate) with direction to the vertical top pillars of the cactus design, using 3 mm/s as scanning speed which is the mark speed of the galvo scanner. The power of the printing process was measured using a digital power meter before the galvo scanner system and set at the value of 100 mW. The intensity at the focal spot was calculated using the formula $I_p = \frac{2P_aT}{Rw_o^2\pi\tau}$ [13] in TW/cm^2, where P_a is the average power before the objective, T the objective's transmittance, R the pulse repetition rate, τ the pulse duration and w_0 the radius of the beam at the beam waist. Here, the values of each pararemer are P_a = 50 mW (50% of power is lost through the galvo scanner and the 4f focusing system), T = 88%, R = 80 MHz, w_0 = 0.5 μm and τ = 150 fs which results in I_p = 1.867 TW/cm^2 at the focal spot. The fabrication process could not be observed since Silicon substrates are not transparent in optical wavelengths,

making the process challenging.

S 8 Characterization

In order to detect the response of the fabricated sample in THz frequencies, Time Domain Spectroscopy (TDS) was used. The electric signal was obtained using photoconductive antennas (PCAs). A linearly polarized THz pulse is generated from the Emitter which is placed at angle $\theta = 0^O$ compared to the incident plane. The detector was placed behind the silicon substrate. The detector receives an electric signal in time domain and using Fourier transformation we get the intensity of the optical response in frequency domain (Figure S10). Depending on the orientation of the sample, we excite the TE and TM mode accordingly by switching the direction that the asymmetry takes place (Figure S11). Measurements were focused on the direction that the magnetic field is perpendicular to the asymmetric part of the metamaterial in order to excite the TM mode which creates the strong EIT phenomenon.

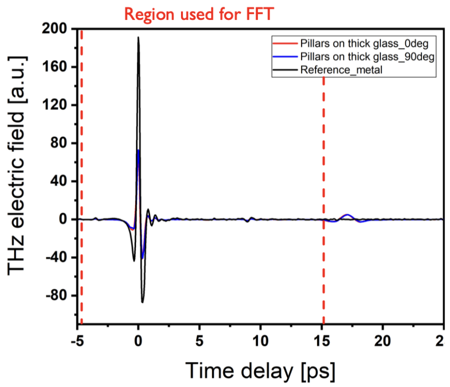

Figure S10: Electric signal detected from PCA in time domain.

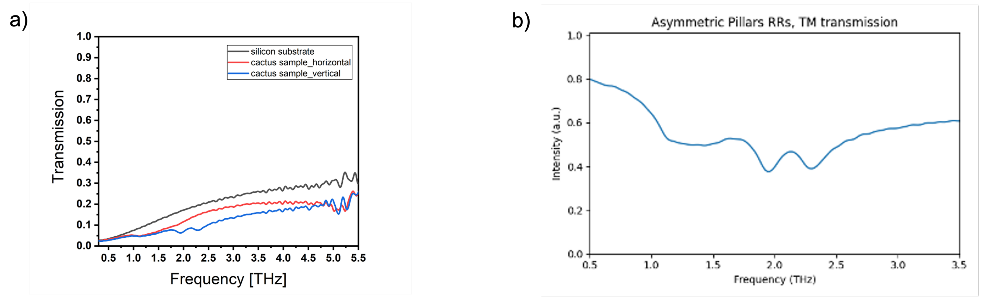

Figure S11: (a) Experimental results of transmission power for different orientation and bare substrate and (b) transmission power results for linearly polarized light along y axis.

S 9 Silver Electroless Plating

The process consists of three steps: seeding, reduction and silver plating, following the protocol described in Ref.[14] The final step of the process was repeated 5 times, to get the required thickness of silver nanoparticles onto the polymer. In Figure S12 the structure before and after SEP is presented.

1. Ag seeding: The sample is immersed in $0.05M$ $AgNO_3$ in H_2O for 38-42 hours. This creates pores on the surface of the polymerized material. Subsequently, the sample is immersed in distilled water and let it dry completely.

2. $NaBH_4$ reduction: Immersing the sample in $6.6M$ $NaBH_4$ aqueous solution for 22.5 hours. In this step the surface is prepared so the Ag nanoparticles can be attached on the polymer. This is a violent chemical interaction, so if the fabricated structures have a big ratio of base and total volume then it is suggested to prepare the solution from the previous day. Subsequently, immerse the sample in distilled water and let it dry completely.

3. Ag plating: In the final step there are three solutions that have to be prepared; (a) 0.2M $AgNO_3$ in H_2O, (b) 5.6% NH_3 in H_2O, (c) 1.9M glucose in H_2O at volume ratios 5:3:8, at room temperature. First, (b) is poured in (a) drop-by-drop until the solution becomes completely transparent. Then (c) is also added to the solution. Finally, the

sample is immersed in the solution for 1-2 minutes, until it starts to getting darker. This step is to cover the surface with densely packed silver nanoparticles. The sample is removed and rinsed with distilled water and let dry completely.

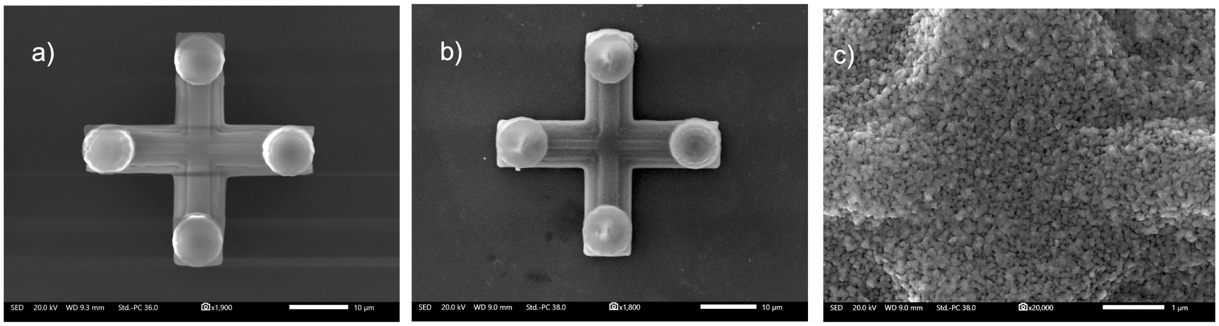

Figure S12: SEM images of the fabricated structure (a) before and (b) after SEP process. In (c) the formed silver nanoparticles are exhibited.

In Figure S12(a) top view SEM image of the "cactus" metamaterial is presented before metallization process. Following, in Figure S12(b) the structure is now metallized and covered with silver nanoparticles, without affecting significantly the Silicon substrate. In Figure S12(c) the aggregates of silver nanoparticles that have coated the polymer's surface is observed. The surface of the polymer is fully coated with silver, resulting in a metallo-dielectric metamaterial structure. After modifying the protocol for SEP in order to be suitable for 0.5-3.5 THz, we complete the study by conducting EDX and conductivity measurements.

9.1 EDX measurements

In order to highlight the selectivity of SEP, the sample underwent through Energy Dispersive X-ray Spectroscopy (EDX). In Figure S13 we observe the diagram that shows the presence of the silver when we target the fabricated-metallized structure. The spot size that EDX measurements were performed was 1 μm. Landing voltage was set at 20 kV, at the scanning electron microscopy system (SEM, JSM-IT700HR InTouchScope^{TM}, Japan). The samples were not sputter-coated since silver is highly conductive and suitable for SEM imaging. Other important elements can also be addressed in larger skin depth through EDX such as Si and Zr which are the basic components of the photoresist that was used.

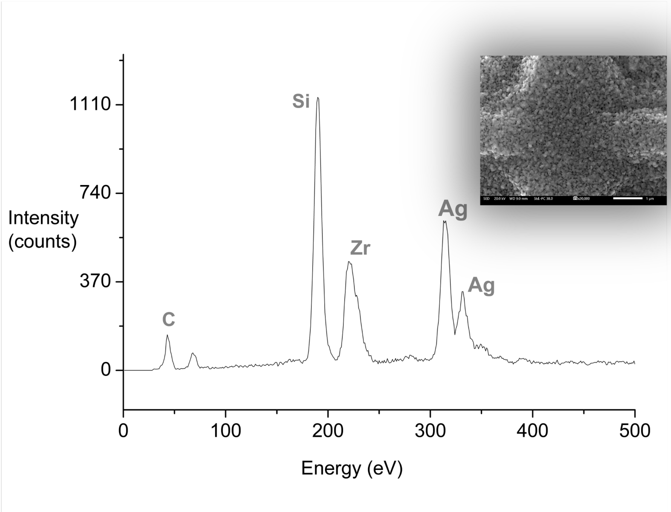

Figure S13: EDX measurement of the sample showing the presence of Silver nanoparticles and zoomed SEM image of the sample.

S 10 Conductivity measurement

After fabrication process and SEP with a modified protocol, SEP was used in cubic geometries (3x3 mm^2) made with MPL, with discrete lines in hatching distances in order to create a woodpile-like structure as it is shown in Figure S14 and get the conductivity results.

Conductivity was calculated by the formula $\sigma = \frac{L}{R*A}$ S/m, where L is the length of the

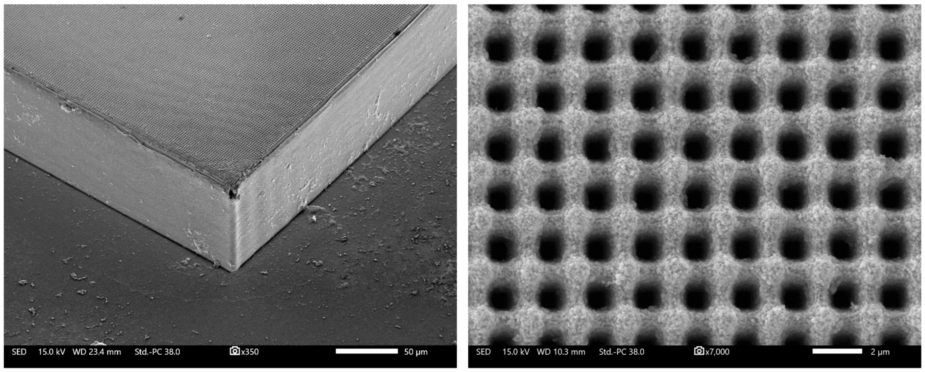

Figure S14: SEM images of fabricated and metallized cubic woodpile-like structures.

area in m, R the measured resistance in Ω and A the cross section of the silver coating in m^2. First we had to measure the resistance of the sample, using microneedles to perform electrical measurements of voltage when we supplied different values of current. Resistance was measured using the formula $R = \frac{AVG(AV)}{AVG(AI)}$ and found to be $R = 2.399\ \Omega$. Length of the area and the cross section was set at $L = 3 * 10^{-3}\ m$ and $A = 2.176 * 10^{-9}\ m^2$ (estimated using multiple SEM images with different magnification), accordingly which resulted in a conductivity value of $\sigma = 5.75 * 10^5\ S/m$.

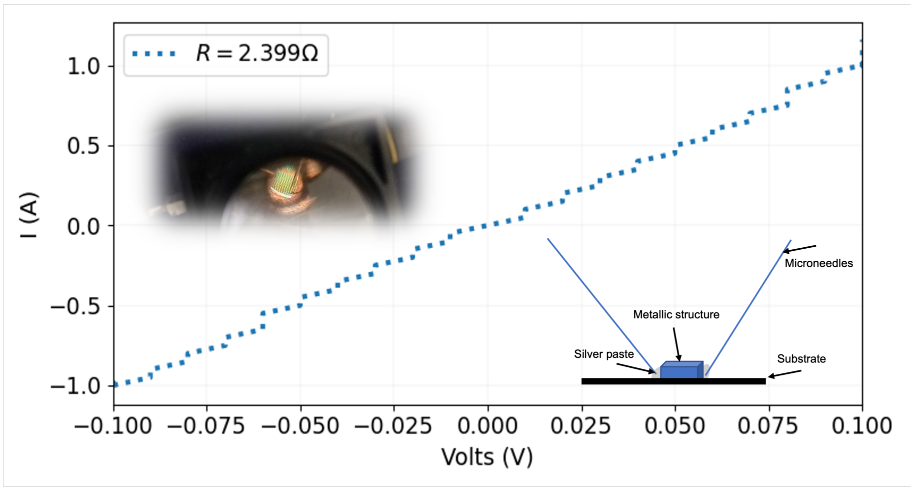

Figure S15: I-V characteristic measurement of the sample. Resistance was calculated from the slope of I-V values. In bottom right corner there is a schematic presentation of the experimental setup for the conductivity measurements.

References

(1) Christopoulos, T.; Tsilipakos, O.; Sinatkas, G.; Kriezis, E. E. On the calculation of the quality factor in contemporary photonic resonant structures. *Optics Express* **2019**, *27*, 14505.

(2) Niemi, T.; Karilainen, A. O.; Tretyakov, S. A. Synthesis of polarization transformers. *IEEE Transactions on Antennas and Propagation* **2013**, *61*, 3102 – 3111.

(3) Asadchy, V. S.; Díaz-Rubio, A.; Tretyakov, S. A. Bianisotropic metasurfaces: Physics and applications. *Nanophotonics* **2018**, *7*, 1069 – 1094.

(4) Ovsianikov, A.; Viertl, J.; Chichkov, B.; Oubaha, M.; MacCraith, B.; Sakellari, I.; Giakoumaki, A.; Gray, D.; Vamvakaki, M.; Farsari, M., et al. Ultra-low shrinkage hybrid photosensitive material for two-photon polymerization microfabrication. *ACS nano* **2008**, *2*, 2257–2262.

(5) Farsari, M.; Vamvakaki, M.; Chichkov, B. N. Multiphoton polymerization of hybrid materials. *Journal of Optics* **2010**, *12*, 124001.

(6) Schroeder, W. F.; Asmussen, S. L.; Cook, W. D.; Vallo, C. I. Efficiency of 4, 4-bis (N, N-diethylamino) benzophenone for the polymerization of dimethacrylate resins in thick sections. *Polymer international* **2011**, *60*, 1362–1369.

(7) Ladika, D.; Noirbent, G.; Dumur, F.; Gigmes, D.; Mourka, A.; Barmparis, G.; Farsari, M.; Gray, D. Synthesis and application of triphenylamine-based aldehydes as photo-initiators for multi-photon lithography. *Applied Physics A* **2022**, *128*, 745.

(8) Sakellari, I.; Kabouraki, E.; Karanikolopoulos, D.; Droulias, S.; Farsari, M.; Loukakos, P.; Vamvakaki, M.; Gray, D. Quantum dot based 3D printed woodpile photonic crystals tuned for the visible. *Nanoscale Advances* **2019**, *1*, 3413–3423.

(9) Deubel, M.; Von Freymann, G.; Wegener, M.; Pereira, S.; Busch, K.; Soukoulis, C. M. Direct laser writing of three-dimensional photonic-crystal templates for telecommunications. *Nature materials* **2004**, *3*, 444–447.

(10) Wang, H.; Zhang, W.; Ladika, D.; Yu, H.; Gailevičius, D.; Wang, H.; Pan, C.-F.; Nair, P. N. S.; Ke, Y.; Mori, T., et al. Two-photon polymerization lithography for optics and photonics: fundamentals, materials, technologies, and applications. *Advanced Functional Materials* **2023**, *33*, 2214211.

(11) Ostendorf, A.; Chichkov, B. N. Two-photon polymerization: a new approach to micromachining. *Photonics spectra* **2006**, *40*, 72.

(12) Ho, K. M.; Chan, C. T.; Soukoulis, C. M.; Biswas, R.; Sigalas, M. Photonic band gaps in three dimensions: New layer-by-layer periodic structures. *Solid State Communications* **1994**, *89*, 413–416.

(13) Skliutas, E.; Lebedevaite, M.; Kabouraki, E.; Baldacchini, T.; Ostrauskaite, J.; Vamvakaki, M.; Farsari, M.; Juodkazis, S.; Malinauskas, M. Polymerization mechanisms initiated by spatio-temporally confined light. *Nanophotonics* **2021**, *10*, 1211–1242.

(14) Katsantonis, I.; Manousidaki, M.; Koulouklidis, A. D.; Daskalaki, C.; Spanos, I.; Kerantzopoulos, C.; Tasolamprou, A. C.; Soukoulis, C. M.; Economou, E. N.; Tzortzakis, S.; Farsari, M.; Kafesaki, M. Strong and Broadband Pure Optical Activity in 3D Printed THz Chiral Metamaterials. *Advanced Optical Materials* **2023**, 2300238.
